# Reelin expression during embryonic development of the pig brain

**DOI:** 10.1186/1471-2202-11-75

**Published:** 2010-06-15

**Authors:** Karsten B Nielsen, Annette Søndergaard, Marianne G Johansen, Kirsten Schauser, Morten Vejlsted, Anders L Nielsen, Arne L Jørgensen, Ida E Holm

**Affiliations:** 1 Institute of Human Genetics, University of Aarhus, Bartholin Building 1240, Wilhelm Meyers Allé, 8000 Aarhus C, Denmark; 2 University of Copenhagen, IBHV, Groennegaardsvej 7, 1870 Frederiksberg C, Denmark; 3 Department of Pathology, Aalborg Hospital, Aarhus University Hospital, Aalborg, Denmark

## Abstract

**Background:**

Reelin is an extracellular glycoprotein of crucial importance in the developmental organisation of neurons in the mammalian cerebral cortex and other laminated brain regions. The pig possesses a gyrencephalic brain that bears resemblance to the human brain. In order to establish an animal model for neuronal migration disorders in the pig, we have studied the expression pattern and structure of Reelin during pig brain development.

**Results:**

We determined the sequence of pig Reelin mRNA and protein and identified a high degree of homology to human Reelin. A peak in Reelin mRNA and protein expression is present during the period of major neurogenesis and neuronal migration. This resembles observations for human brain development. Immunohistochemical analysis showed the highest expression of Reelin in the Cajal-Reztius cells of the marginal zone, in resemblance with observations for the developing brain in humans and other mammalian species.

**Conclusions:**

We conclude that the pig might serve as an alternative animal model to study Reelin functions and that manipulation of the pig Reelin could allow the establishment of an animal model for human neuronal migration disorders.

## Background

The mature cerebral cortex of higher mammals consists of a six-layered structure with each layer comprising different types of neurons that perform distinct functions. The layered structure is created during development by migration of young post-mitotic neurons along glial fibers from the proliferative zones lining the ventricles to the outer layers of the developing cortical plate (CP). The post-mitotic neurons follow an ordered inside-out pattern of migration were young neurons migrate past their predecessors to their correct position in more superficial layers of the CP [1,2]. Proper cortical layering calls for highly synchronized processes of neuronal genesis, migration and maturation. The mechanisms controlling the whole-cell movement are well understood, but knowledge of how the neurons are instructed to leave their glial guidance upon reaching their destination remains unclear.

Genetic studies of human malformation and mutant mice with deficient neuronal migration have played an integral role in unveiling the existence of signalling pathways that regulate neuronal migration and neuronal detachment from the glial fibers [3,4]. The Reelin pathway is one of the most intensively studied pathways involved in neuronal migration. Mutations in the Reelin gene is associated with neuroanatomic abnormalities in reeler mice [5,6] and causes recessive lissencephaly with cerebellar hypoplasia in humans [7,8]. The reeler phenotype is characterized by disordered cellular positioning of neurons in the major laminated structures of the brain, including the cerebral and cerebellar cortices and hippocampus [4,5,9,10]. The post-mitotic neurons derived from the radial progenitors in the subventricular zone begin their migration at the normal developmental stage and proceed as far as the intermediate zone. The newborn neurons, however, fail to split the preplate and the subsequently generated neurons accumulate underneath the previously generated neurons, forming a scattered neuron distribution with an approximately inverted cortical plate (outside-in) [5,10,11].

Reelin is a large extracellular glycoprotein known to influence migrating neurons as an initiator of a pathway that mediates a signal from the extracellular matrix. The signal transduction is accomplished through direct binding to the ectodomains of the lipoprotein receptors Very Low Density Lipoprotein Receptor (VLDLR) and Apolipoprotein Receptor 2 (ApoER2) [12,13] upon binding to the receptors a rapid phosphorylation of the cytoplasmic adapter protein Disabled-1 (Dab1) is induced by members of the Src family tyrosine kinases [14-18]. Dab1 phosphorylation is succeeded by cytoskeletal rearrangements [17,18]. Cytoskeletal rearrangements are accomplished through direct interactions between phosphorylated Dab1 and a variety of proteins involved in actin remodeling such as neuronal Wiskott-Aldrich syndrome protein and Rap-GTP and Lissencephaly-1, nuclear distribution gene E homolog like 1, Tau, and microtubule-associated protein 1B in microtubule reorganization [12,19-21]. Mutations in mouse Reelin, ApoER2 and VLDLR (double receptor mutant) or Dab1 result in highly similar phenotypes with the inability of neurons to split the preplate and inability of glial-guided neurons to bypass their predecessors [19,22]. The mechanism by which the Reelin pathway facilitates correct neuronal positioning remains unclear although Reelin has been suggested to control detachment of migrating neurons from radial-glial cells [23,24]. Recent data indicate that another function of Reelin could be to activate Notch [25]. Interaction between phosphorylated Dab1 and the activated form of Notch (Notch intracellular domain; NICD) inhibits polyubiquitination and degradation of NICD, which is expected to strengthen Notch signaling [25,26].

Expression analysis of Reelin during development has been studied in a wide range of animals spanning from reptiles over birds to mammals including mice and human [27-32]. These studies indicate that the Reelin-expressing cells are conserved through evolution and Reelin has played a critical role in cortical evolution. In mammals, Reelin has the highest expression in Cajal-Retzius (CR) cells during neocortical development [5,6,10,30,33,34]. CR cells appear early in development and constitute the majority of the neurons in the marginal zone (MZ) [35] but they are transient neurons and disappear between postnatal week 2 and 3 in rodents and around gestational week 27 in humans [36,37]. In humans, CR cells have characteristic morphological appearance with a rich axonal arborization orientated horizontally [32,38]. In rodents, CR cells have a more uniform morphology and can be identified based on three morphologic criteria: 1) usually bipolar with 2) horizontal orientation and 3) fusiform or ovoid shape [37,39]. In pigs, CR cells resemble other mammalian CR with respect to localization in the outer part of MZ, orientation parallel to the pial surface, and bipolar and fusiform somata [40,41].

The domestic pig has emerged as an attractive non-primate animal model for studies of brain development, due to the similarity between human and pig embryonic brains [42]. The gyrencephalic pig brain bears resemblance to the human brain with respect to gross anatomy, cytoarchitecture, size and development rather than to the brains from small rodent laboratory animals [42]. In this study we have identified and characterized pig Reelin and determined the expression pattern of Reelin during embryonic cortical development in this species.

## Results and Discussion

### Sequence of the pig Reelin mRNA and protein

**Figure 1:**
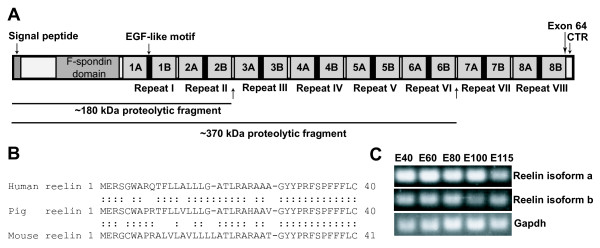
**Characterization of pig Reelin**. A) Schematic drawing of the pig Reelin protein. The localization of the signal peptide and the conserved CTR are indicated. The eight Reelin repeats with subdomains A and B are shown together with the localization of the EGF- repeat like subdomain. An arrow indicates the position of the alternative splices Reelin exon 64. The position of proteolytic sites in Reelin from human and a corresponding peptide sized approximately 180 kDa and 370 examined by western blotting shown beneath the sequence. B) Sequence homology analysis of the Reelin signal peptide from mouse, pig and human. Aminoacids 1 to 40 are shown. Gaps for alignment are indicated by (-). Conserved aminoacids are indicated by (:). C) Detection of alternative splicing of pig Reelin. Primers were designed specific for Reelin isoform a including exon 64 (upper panel); specific for Reelin isoform b excluding exon 64 (central panel); or GAPDH as internal control. Expression was detected by reverse transcriptase PCR with cortical cDNA from the indicated time points.

As a first step towards characterization of pig Reelin we determined the sequence of mRNA and protein. As the pig genome sequence is not available, the Reelin mRNA sequence was assembled from combined *in silico *and RT-PCR analysis. A 11,694 bp mRNA sequence was determined including an open reading frame from position 283 to 10,665 with potential to encode a protein consisting of 3460 amino acids [Genebank accession no. BK006770]. The deduced molecular weight of the pig Reelin protein is 388 kD, not taking into account post translational modifications such as glycosylation. Protein alignment to human Reelin showed 96% identity and 98% similarity. Notably, no gaps were introduced in the alignment of the pig and human Reelin proteins. Protein alignment to mouse Reelin showed 94% identity and 98% similarity with two gaps. Like the human and mouse Reelin proteins the pig Reelin includes 8 Reelin-repeats of 350-390 amino acids containing a pattern of conserved residues related to epidermal growth factor (EGF) repeats (Figure 1A). The Reelin repeats can be divided into two sub-repeats A and B (see Figure 1A) [43]. The C-terminal region (CTR) of Reelin, consisting of 32 amino acids, is highly conserved between species, and the pig Reelin indeed includes such a completely conserved CTR. Another characteristic of Reelin is the existence of a 27 amino acid signal peptide in the N-terminus. Protein alignment of the N-terminal region showed a more pronounced similarity between human and pig Reelin than between mouse and pig Reelin (Figure 1B). Whether this difference in conservation has biological implications needs further investigation.

The human and mouse Reelin genes consist of 65 exons and our analysis indicates a conserved genomic structure of the pig Reelin gene (data not shown). Evolutionary conserved alternative splicing of a brain specific Reelin gene miniexon, exon 64, has been described [44]. Human and mouse Reelin gene exon 64 consists of 6 nt. The above described pig Reelin mRNA includes a 6 nt exon 64 and according to the nomenclature of human and mouse Reelin the corresponding pig mRNA is abbreviated isoform a. To determine if Reelin isoform b, which lacks exon 64 in the mRNA sequence, also existed in the pig we performed a RT-PCR analysis. Two different forward primers were designed which could distinguish the inclusion of exon 64 and the primers were used in RT-PCR together with a reverse primer in exon 65 (Figure 1C). As template was used pig embryonic cortical cDNA. By this approach we could detect the existence of Reelin isoform b [Genebank accession no. BK006771]. Using cortical cDNA representing different developmental points we observed parallel expression profiles for the two Reelin isoforms in accordance with observations from other mammalian species [44]. Using a RT-PCR primer combination from exon 63 to exon 65 followed by sequencing we estimated that pig Reelin isoform b was expressed at a lower level than isoform a, also in accordance with data from other mammalians (data not shown) [44]. In summary, pig Reelin mRNA and protein show highly conserved properties compared to other mammalian species.

### Expression of pig Reelin cDNA and protein in the developing cortex

**Figure 2:**
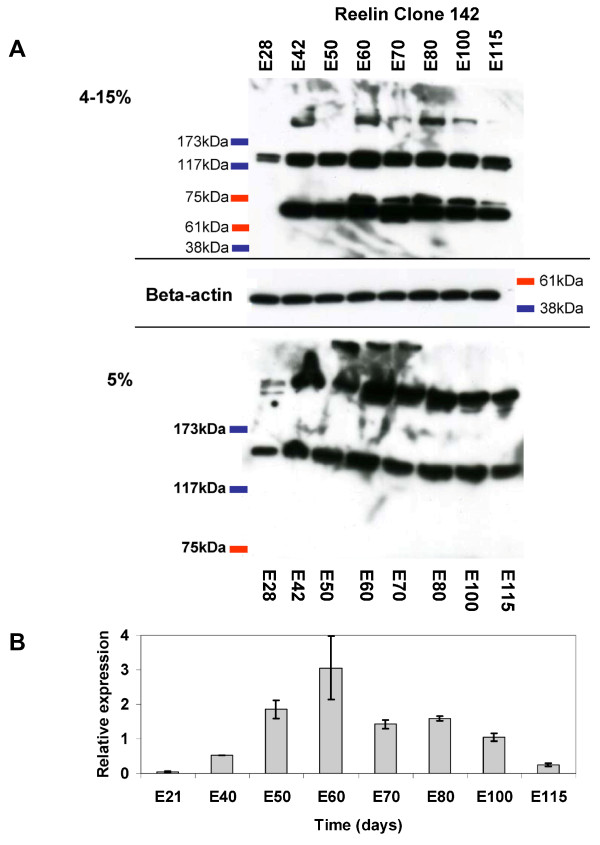
**Expression analysis of pig Reelin during development**. A) Western blot analysis with the monoclonal anti-Reelin antibody Clone 142 on protein samples purified from cerebral cortex of embryonic pigs at the indicated developmental stages. Brain extracts were resolved by PAGE using either a 5% (lower) or 4-15%-gradient gel (upper). Three bands are seen in the samples, of about 400, 320 and ~160 kDa in the 5% gels and 320, ~160, 70, and 65 kDa in the gradient gels. Beta-actin was used as loading control. B) Quantitative analyses of Reelin mRNA expression during pig embryonic brain development. The expression levels were normalized to the expression levels of GAPDH, 18S rRNA and β-actin in the same tissue using the geNorm program.

We studied the expression pattern of Reelin protein during pig brain development by Western blot analysis. A Reelin protein band was clearly visible at the early stage, E28, which corresponds to the phase of preplate formation in the pig (Figure 2A). The Clone 142 antibody detects the full-length protein band in the 5% gel (Figure 2A, lower image) in the lanes containing cortical extracts from E50, E60 and E70. The presence of this band is restricted to this time interval. The other bands recognized in the 5% gel correspond to length of around 320 kDa and 160 kDa, which are the dominant bands in extract from brain tissue [45,46].

In 4-15% gradient gels no clear full length band was detected. It may be trapped in the slot due to high contend of polyacrylamid fibers. The 320 kDa band appeared between E42 and E100 while two strong bands of about 160 kDa and 65 kDa in length were consistently present except for the 65 kDa band at E28. The low molecular band of 65 kDa has been observed in extracts from brain tissue as far back in phylogeny as sea lamprey using both clones 142 and G10 Reelin antibodies. It has been suggested to represent a proteolytic fragment [47], but there is no identified proteolytic site in the N-terminal region of Reelin recognized by the antibodies that could account for the size of 65 kDa corresponding to, approximately, 650aa.

Interestingly, a band of ~70 kDa was detected at very low intensity at E50, growing in intensity to peak in E80 and then decreasing (Figure 2A, 4-15% gel). This band has not previously been described in any animal. The intensity variation does to some extend follow the bell-shaped-like variation in RNA expression of the Reelin gene during this embryonic period (Figure 2B). When comparing the intensities of the other bands in the gel the ~70 kDa band does not appear to be a proteolytic product of either the 320 kDa or 160 kDa bands. An alternative explanation could be a new splice variant found in the porcine brain. Western blotting experiments performed on mouse brain tissue spanning the period from embryonic day 9 to postnatal day 14 did not reveal this band (data not shown). A functional implication of the ~70 kDa band is difficult to predict since Jossin *et al*. showed that a protein fragment consisting of the repeats 3 to 6 is necessary and sufficient for binding to the lipoprotein receptors and subsequent phosphorylation of dab1 [48].

The Reelin expression gradually increased to reach a peak of expression at E60, corresponding to a period of intense neuronal migration form the proliferative layers into the cortical plate (Figure 2A). The Reelin expression slightly decreased through the late developmental stages (Figure 2A).

mRNA expression levels were analyzed by qRT-PCR and the resulting pattern was consistent with the observations from Reelin immunoblots (Figure 2A and 2B). mRNA was initially detected at very low levels at E21 (Figure 2B). This stage corresponds to an early stage in preplate formation. The mRNA levels increased from E21 through E40 and E50 to a peak at E60. This increase was followed by a decline in expression to a plateau level from E60 to E100 followed by a further decrease to E115 (Figure 2B).

### Immunohistochemical examination of pig Reelin expression in the developing cortex

**Figure 3:**
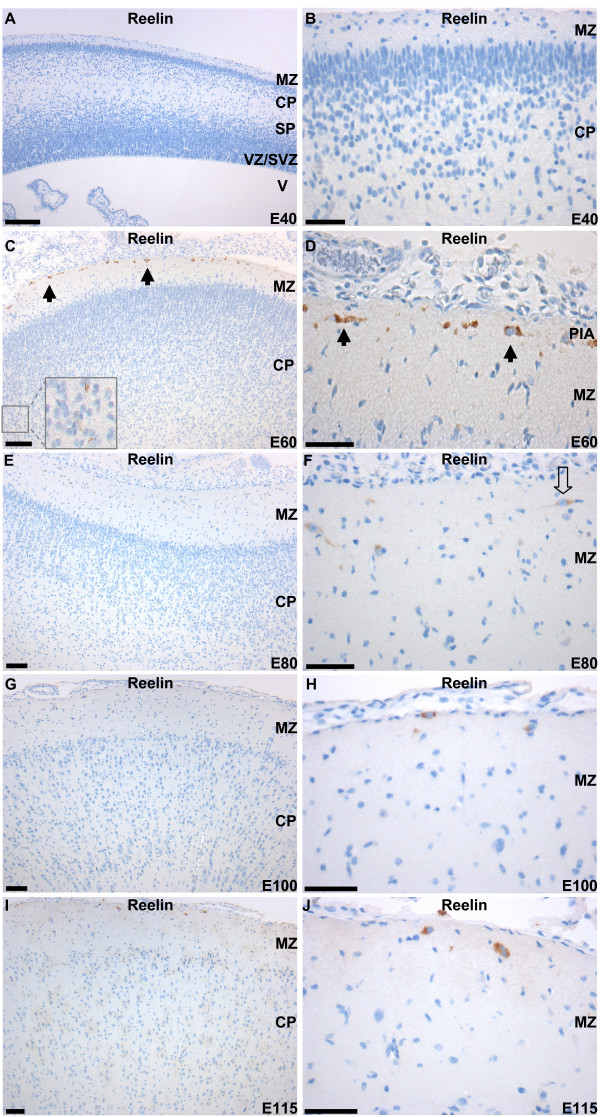
**Immunohistochemical staining for Reelin in pig neocortex from five embryonic stages**. A and B) Immunohistochemical staining of porcine cortex at E40. No staining was observed. C and D) Immunohistochemical staining of cortex at E60. Reelin expression is identified in CR cells of the marginal zone (MZ). B) Immunohistochemical staining at E60 at higher magnification. Black arrows indicate horizontal Reelin positive CR cells. Reelin immunoreactive interneurons in layer III-IV of the cortical plate is magnified (x10) in the box. E and F) Immunohistochemical staining at E80. Reelin expression is detectable with decreased intensity in the cortex. Bipolar fusiform CR type cells with dendrites oriented parallel with the pial surface were observed in the outer part of the MZ (open arrow) along with Reelin-immunoreactive interneurons and CR type cells displaying a variety of shapes and sizes. Reelin positive interneurons are seen in the CP but in decreased numbers. G and H) Immunoreactivity is observed in the cytoplasm of CR in the MZ at decreased numbers. The Reelin expression in the interneurons of the cortical plate is no longer detectable. I and J) Reelin positive cells are detectable in the outer part of MZ at E115. Scattered immunoreactivity is observed in the extracellular matrix throughout the CP. Cortical plate: CP; Marginal zone: MZ; pia mater: PIA; subplate: SP; VZ; subventricular zone: SVZ; 3^rd ^ventricle: V; ventricular zone: VZ. Scale bar = 100 μm.

Immunohistochemical staining for Reelin at E40 (and earlier time points; data not shown) showed no staining of any cell type (Figure 3A and 3B). At E60, Reelin positive cells were seen in the outer part of the MZ (Figure 3C and 3D) and a few small round immunoreactive cells were identified in layers III-V of the cortical plate (CP) (Fig 3C and small magnification image). These cells were identified as interneurons in a variety of mammals [32,49,50]. For the most part, the antibody against Reelin stained the cytoplasm of the cells and immunoreactive dendrites and axons were rarely observed. The Reelin expressing neurons located in the outer part of the MZ displayed a different morphology. The main part of the neurons were elongated with the axis oriented parallel to the pial surface, resemble CR cells (Figure 3D), others were small round interneurons (Figure 3D) and neurons oriented perpendicular to the pial surface could be indentified in the inner part of the MZ. At later stages E80, E100, and E115 (Figure 3E to 3J), the immunoreactive cells decreased in number. A few bipolar and fusiform CR type cells restricted to the outer part of the MZ could be identified (indicated with open arrow in Figure 3F; see also Figure 3H and 3J) along with a decreasing number of Reelin positive interneurons in the CP at E80 (Figure 3E) these neurons were not detectable at later embryonic stages.

## Conclusion

In the present study, we describe the identification and characterization of pig Reelin cDNA and protein. The Reelin protein shows a high degree of homology to the human Reelin. The expression profile of pig Reelin during cortical development resembles human Reelin. In accordance with the mRNA expression level the Reelin protein expression is initiated at early developmental stages and increases during corticogenesis. The same expression patterns are reported in human and the mouse embryonic brain tissue [31,32]. Immunohistochemical analysis of Reelin expression also shows localization within CR cells of the MZ of the developing cortex similar to human Reelin. The Reelin characterization described here support the potential for establishing the pig as a model for human neuronal migration disorders.

## Methods

### Tissue

Embryos/foetuses were obtained from artificially inseminated landrace sows (Sus Scrofa). Pregnant sows were anaesthetized by inhalation of 35-70% CO_2 _for one minute and sacrificed by exsanguination. The uteri were recovered immediately and the embryos/foetuses from 21, 28, 40, 50, 60, 70, 80, 100, and 115 days post conception (dpc/E) were quickly removed, and dissected. The anterior part was dissected form 21 and 28 embryos. Cortices from the remaining tissue were dissected and flash frozen in liquid nitrogen immediately after dissection and stored at -80°C after dissection. Tissue for immunohistochemical analysis was immersed in formalin (Sigma-Aldrich). All experiments were approved by the local and national animal ethical committee and are in accordance with National legislation on experimental animals.

### Immunohistochemistry

Formalin fixated tissues were embedded in paraffin blocks and cut in 4 μm sections and mounted on superfrost slides. Slides were dewaxed in xylene and rehydrated using graded ethanol washes. All immunohistochemistry was performed using the automated staining system Autostainer (DAKO, Glostrup, Denmark). Sections were stained with antibodies directed against Reelin (1/400; Chemicon, MAB5366). IHC staining was performed using DakoCytomation EnVision + System HRP (#K4004) according to the manufacturer's instructions. Sections were counterstained with hematoxylin for 30 s, dehydrated in graded ethanol washes, and mounted. Human brain tissue (normal and tumour tissue) was used as control. Microscopic images were taken with a DC 480 camera (Leica Microsystems A/S) and processed using IM50 imaging and capture software (Leica Microsystems A/S).

### Western blotting

100-200 mg of tissue representing the entire cortical sheet from flash frozen frontal cortex was transferred to a Dounce tissue-grinder (Wheaton #357542). 2 ml Lysis buffer (Tris-HCl 50 mM, NaCl 150 mM, 1% NP-40, one Protease Inhibitor Cocktail Tablet (Roche)/10 ml Lysis buffer; pH 8.0) was added and homogenisation was performed on ice [51]. Solution was transferred to Eppendorf tubes (1 ml) and centrifuged at 20,000 rpm for 20 min. (4°C). The supernatant was transferred to new tubes. The protein extract was kept on ice until use.

Protein concentrations were measured using by the Bradford method.

Four micrograms of protein was added 5 × Loading Buffer (Fermentas), 20 × Reducing Agent (Fermentas) and PBS to a final volume of 15 μl and a 1 × concentration, and the samples were heated to 100°C for 2 min. The time of denaturation at 100°C was reduced to 2 min. to reduce degradation of the full-length Reelin protein. Western blotting analyses were performed at standard conditions using alkaline phosphatase-conjugated secondary antibodies for detection and visualization by BM Chemiluminescence (Roche). Beta-Actin (Abcam, #ab8227) was used as loading control (1:10,000) and the monoclonal Reelin (clone 142; Calbiochem, # 553730) antibody was used. Samples were separated on 4-15% Tris-HCl gels (Bio-Rad), run in parallel with ProSieve Color Protein Markers (Lonza, # 50550), and transferred to PVDF membranes (HYBOND-P PVDF 0.45 μm pore size; GE Helthcare, # 25006203). Blots were blocked in blocking buffer (10% non-fat dry milk, 1% Tween 20 and PBS), overnight at 4 degrees, rinsed, incubated in primary antibody (diluted in 0,5% non-fat dry milk, 0,1% Tween 20 and PBS) for 1.5 hr (all steps at room temperature), rinsed, incubated in peroxidase-conjugated secondary antibody in blocking buffer for 1 hr, rinsed, incubated in enhanced BM Chemiluminescence (Roche, # 11500694001), and exposed to film (Hyperfilm MP; GE Helthcare, # 25800086) for 0.1-10 min.

### RNA extraction, cDNA synthesis, and RT-PCR

Unless otherwise stated, all solutions were prepared with water that had been treated with 0.1% diethyl pyrocarbonate (DEPC). Tissue (50-100 mg) representing the entire cortical sheet was dissected from frontal cortices and homogenized in an RNase-free Dounce tissue-grinder (Wheaton #357542). 2 ml TriReagent (Sigma) was added and homogenisation was performed. RNA was extracted using chloroform and Isopropanol precipitation. The RNA pellet was washed twice in 70% Ethanol and re-suspended in 50 μl DEPC-H_2_O. The quantity was spectrophotometrically determined by measuring the absorbance at wavelength 260 (OD_260_) and the quality checked by electrophoresis of 0.5 μg RNA on ethidium bromide stained 1% agarose gels.

cDNA was synthesized from 2,5 μg total RNA in 50-μL reactions using iScript™ cDNA synthesis Kit (Biorad). After synthesis, the cDNA was diluted five times with water and stored at 4°C.

RT-PCR analysis to detect Reelin spliceforms were done at standard PCR conditions with 30 cycles of amplification. Primers for the analysis were: Exon65Reverse, GGATTCTTCATGGGTATCG TC; Reelin-a-forward, CATGTGGAAGTTGTCCTAGT; Reelin-b-forward, CATGTGGAAGTTGT CCTCAC.

### Quantitative real-timePCR

qRT-PCR was performed using the iCycler Thermal Cycler (Bio-Rad, iCycler iQ™ Real-time PCR Detection System). Reactions were performed in triplicates in a total volume of 20 μl each using DyNAmoTM HS SYBR^® ^GREEN qPCR Kit (Finnzymes). The primers were designed using primer3 http://frodo.wi.mit.edu/primer3/ with a total length between 18-22 bp, T_m _58 ≤ 60 ≥ 63 and a GC% of min. 40% and max. 80%. Primers were restricted to produce an amplicon in the range of 180 to 220 bp. Pig sequences were used if available. When pig sequences were unavailable, primers were designed from conserved regions among human, mouse and bovine sequences. A BLAST analysis was performed to eliminate unspecific hybridization of homolog sequences. Two micro litres cDNA was used as template and 6 pmol of each primer, designed to amplify across at least one predicted exon-intron boundary (sequences in Table 1). Exon-intron boundaries were predicted using the Ensembl Human Geneview database http://www.ensembl.org/Homo_sapiens/index.html. Primer efficiency was measured by dilution standard curves (efficiency >95%). The amount of mRNA was normalized to the measured expression of three normalizing genes glyceraldehyde 3-phosphate dehydrogenase (GAPDH), Beta-actin and 18S rRNA mRNA in each sample and the quantification was done using the GeNorm program [52]. After each assay, the identity of the PCR products was confirmed by melting-curve analysis, gel electrophoresis and, sequenced to confirm the identity of the PCR amplicon. Primer sequences are presented in Table 1.

**Table 1:** qRT-PCR primer data

Quantitative real-time PCR primers				
Gene	Primer sequences	Annealing temperature (°C)	Amplicon lenght (bp)	Accession number
	Forward: 5'-TGC TGT CAC CTT CTG TGA GC-3'			
Reln	Reverse: 5'-GAT GGT TGT GCT GAC ATT GG-3'	60	217	XM540392
	Forward: 5'-CCA CGA GAC CAC CTT CAA CT-3'			
ACTB	Reverse: 5'-GAT CTT GAT CTT CAT GGT GCT G-3'	60	210	BM190097
	Forward: 5'-CTG ACC GGG TTG GTT TTG AT-3'			
18S rRNA	Reverse: 5'-AGC GAG CGA CCA AAG GAA CC-3'	59	134	NR002170
	Forward: 5'-TCA TCA TCT CTG CCC CTT CT-3'			
GAPDH	Reverse: 5'-TGT GGT CAT GAG TCC TTC CA-3'	58	202	AF017079

## Authors' contributions

KBN carried out the molecular genetic and bioinformatics studies along with western blotting and qRT-PCR experiments and drafted the manuscript. AS and MGJ participated in performing the qRT-PCR and immunohistochemistry experiments (respectively). KS and MV collected the porcine tissue. IEH, ALJ, and ALN participated in the design of the study, in the evaluation of the results, and in drafting and finalizing the manuscript. All authors read and approved the final manuscript.
